# Hemipartial Laminectomy and Bilateral Flavectomy Technique With Unilateral Approach in Patients With Cervical Spinal Stenosis Due to Ligamentum Flavum Hypertrophy: A Technique Note

**DOI:** 10.7759/cureus.20040

**Published:** 2021-11-30

**Authors:** Salim Senturk, Ülkün Ünsal, Serdar Çevik, Onur Yaman

**Affiliations:** 1 Neurosurgery, Memorial Spina Center, Istanbul, TUR; 2 Neurosurgery, Manisa Şehir Hospital, Manisa, TUR; 3 Neurosurgery, Memorial Şişli Hospital, Istanbul, TUR; 4 Neurosurgery, Memorial Hospital, Istanbul, TUR

**Keywords:** ligamentum flavum hypertrophy, hemipartial laminectomy, flavectomy

## Abstract

The aim of this procedure is to widen the spinal canal by using minimally invasive techniques to do hemipartial laminectomy and bilateral flavectomy in patients with cervical spinal stenosis due to ligamentum flavum hypertrophy.

A 66-year-old man presented with increasing neck and right shoulder pain for one year to Koç University Hospital. He reported a three-month history of numbness in his hands. The Japanese Orthopedic Association (JOA) and Visual Analogue Scale (VAS) scores were 15 and 8, respectively. Preoperative magnetic resonance imaging (MRI) revealed spinal canal stenosis at the C3-4 level secondary to ligamentum flavum hypertrophy. Hemi-partial laminectomy at the C3 level, flavectomy, and bilateral decompression were performed using the right unilateral approach. The patient's complaints of symptoms considerably decreased three months later. The VAS and JOA scores were 2 and 16, respectively.

This minimally invasive approach can be an alternative to classic laminectomy in patients who have radiculopathy and myelopathy due to posterior origin spinal stenosis in order to safely resolve pain and neurologic dysfunction.

## Introduction

Introduction

For several years, various techniques have been evaluated to determine the most accurate treatment for cervical spondylosis causing myelopathy and radiculopathy. Even though many studies have been conducted in the last decades, there still is not a consensus on the optimal surgical management. In general, surgical approaches can be divided into anterior, posterior and anterior and posterior cervical canal decompression approaches. Each approach can be supplemented by fusion. The anterior approach typically comprises anterior cervical discectomy with fusion and anterior cervical corpectomy with fusion, whereas laminectomy in the presence and/or absence of instrumentation and/or laminoplasty is included in the posterior approach. Long-term follow-up studies on laminectomy for multilevel cervical stenosis have reported that the procedure causes serious postoperative complications, such as segmental instability, progressive kyphosis, swan-neck deformity, and perineural adhesions; laminoplasty, which is a commonly used procedure for treating cervical stenosis owing to its posterior origin and the hypertrophy or ossification of the ligamentum flavum, was developed to avoid these complications [1-6]. Furthermore, with the more common use of minimally invasive approaches, there has been renewed interest in posterior approaches for the treatment of cervical spine disorders.

This study aimed to introduce a minimally invasive alternative approach for the treatment of cervical stenosis resulting from ligamentum flavum hypertrophy

## Technical report

Surgical technique

**Figure 1:**
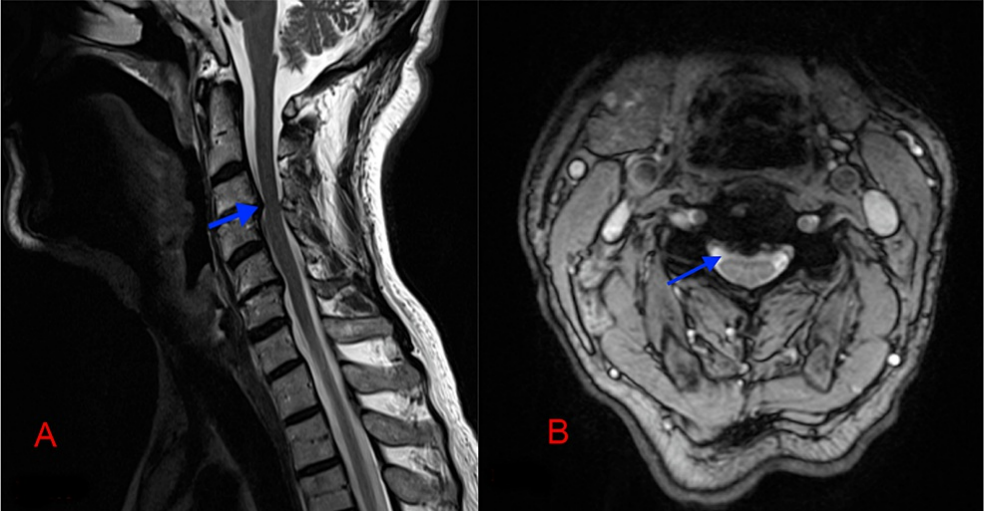
(A) Preoperative MRI T2-sagittal and (B) T2-axial sequences, blue arrows show the C3-4 spine canal stenosis.

A 66-year-old man presented with increasing neck and right shoulder pain for one year at Koç University Hospital. He reported a three-month history of numbness in his hands. The Japanese Orthopedic Association (JOA) and Visual Analogue Scale (VAS) scores were 15 and 8, respectively. Preoperative magnetic resonance imaging (MRI) revealed spinal canal stenosis at the C3-4 level secondary to ligamentum flavum hypertrophy (Figure 1). We planned bilateral decompression with a unilateral approach to decompress spinal stenosis at the C3 level.

**Figure 2:**
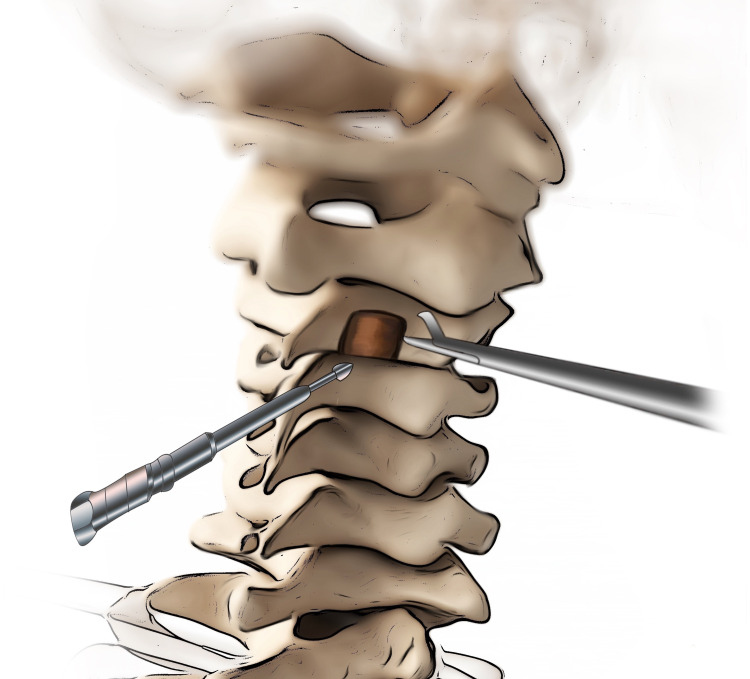
The lamina of the level above the stenotic level was thinned from two-thirds of the inferior side towards the midline without touching the stenotic zone using a drill.

The patient, under the influence of general anesthesia, was placed and fixed to the operation table in the prone position on a crescent headrest, and a linear midline incision followed by a unilateral subcutaneous dissection was made. Fluoroscopic guidance was used to determine the number of levels to be exposed. Two laminae were exposed via subperiosteal dissection. The stenotic level was exposed after verifying the level using fluoroscopy. The lamina of the C3 was thinned from two-thirds of the inferior side towards the midline without touching the stenotic zone using a drill (Figure 2).

**Figure 3:**
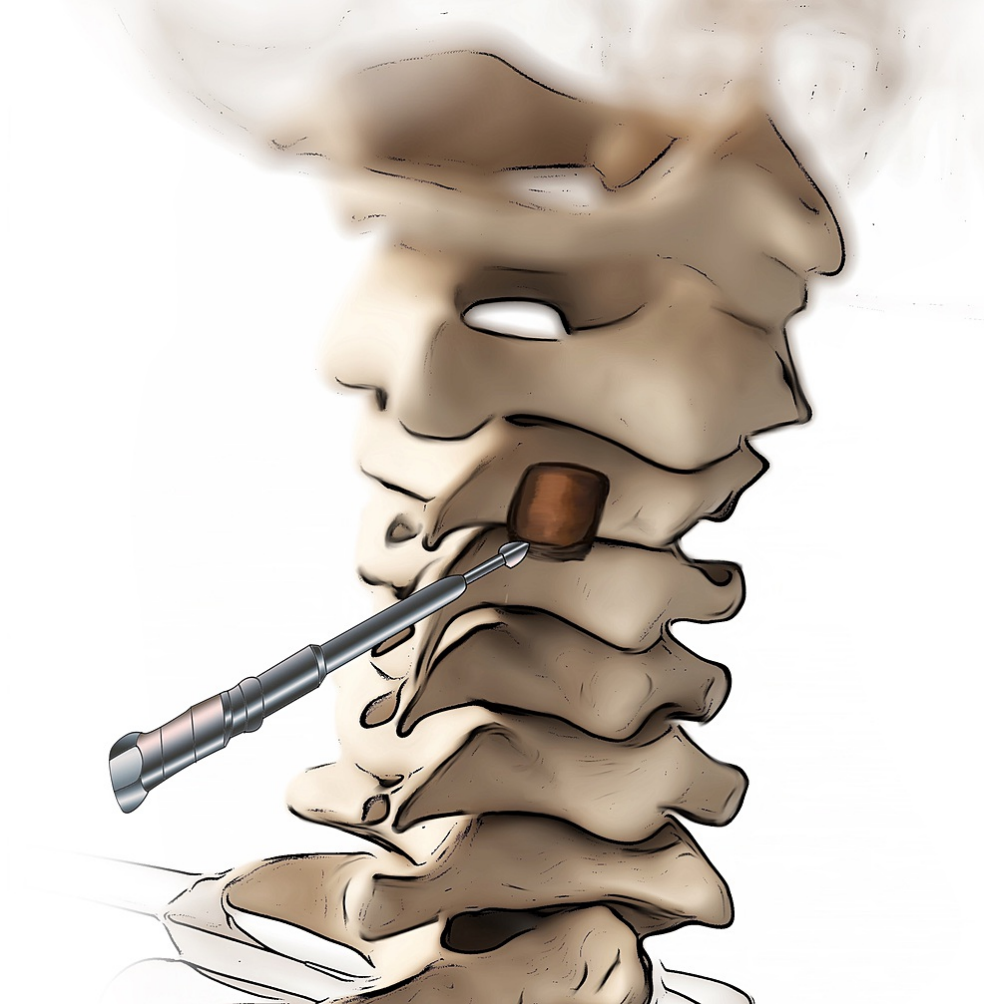
The lamina of the level below the stenotic level was also thinned from one-quarter of the superior side. On the lateral side, the lamina was thinned approximately 2 mm from the lamina mass junction using a drill.

**Figure 4:**
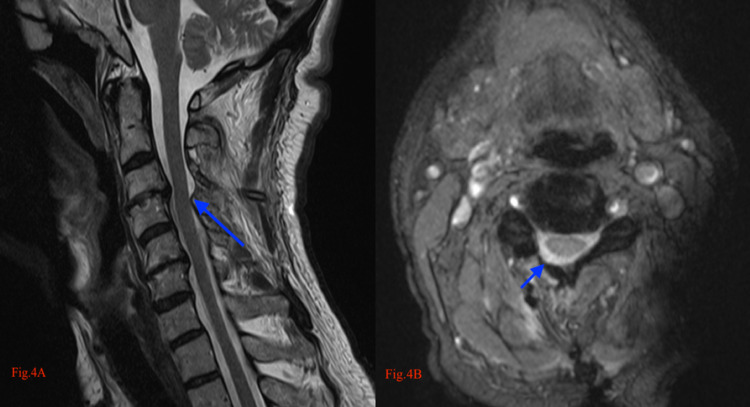
(A) Postoperative MRI T2-sagittal and (B) T2-axial sequences; blue arrows show the region after surgery (ULBD) ULBD: unilateral approach and bilateral decompression

The lamina of the C4 was also thinned from one-quarter of the superior side. On the lateral side, the lamina was thinned approximately 2 mm from the lamina mass junction using a drill (Figure 3). Thus, a rectangular window was created to expose the stenotic zone. Using a 1-mm Kerrison rongeur, the spinal canal was enlarged from the thinned-bone area by removing parts of the lamina and flavum causing compression. The stenotic zone remained untouched. The spinal cord was observed to mobilize toward the decompressed lamina side. A canal was opened by reclining a dissector into the spinous process at the midline. The surgical table and microscope were positioned for bilateral decompression using a unilateral approach. The flavum under the lamina of the other side was excised using a 1-mm Kerrison rongeur and biopsy punch. These procedures bilaterally enlarged the spinal canal. Bilateral decompression was achieved via hemi-partial laminectomy and flavectomy using the unilateral approach (Figure 4). The spinal cord was not manipulated nor mobilized by the surgical instruments or maneuvers during the surgery. The patient was mobilized by the evening of the same day the surgery was performed. The patient was discharged post hemovac drain removal after one day.

## Discussion

Standard cervical laminectomy performed on normal adult patients disrupts the posterior tension band and the cervical spine alignment forces and increases the risk of post-laminectomy kyphosis. This results in an unstable spine and possible post-laminectomy kyphosis or instability [5,7]. Thus, one should focus on preserving the posterior cervical structures as much as possible when developing techniques used in posterior cervical approaches. 

Injury to the extensor muscles is another important condition that leads to posterior spinal deformities (semispinalis cervicis and capitis, multifidus and rotator muscles) [1,8]. The deep extensor muscles of the neck, e.g., semispinalis cervicis and multifidus, are directly attached to the spinous processes, which serve as levers in extensions. These muscles act as major power sources and dynamic stabilizers for cervical spine motion [8,9]. The C2 spinous process, the largest of all cervical spinous processes, contains five bilateral muscle attachments crucial for stabilizing and mobilizing the neck. These muscles are invariably detached from the spinous processes in conventional posterior surgeries of the cervical spine, resulting in postoperative problems, such as persistent neck pain, shoulder stiffness, swan-neck deformity, restricted range of motion, and spinal malalignment [10-14].

Kamioka et al. [15] reported that supra- and inter-spinous ligaments and posterior facet joints contribute to cervical spinal stabilization. According to Nolan and Sherk [8], the neck extensor muscles are important dynamic stabilizers for the cervical spine. Fujimura and Nishi [16] followed up 53 patients for five years and observed an 80% reduction in their cervical muscle diameter; furthermore, one year postoperatively, they observed that the deep cervical muscles had atrophied by 30% compared with that before surgery. In 1999, Hidai et al. [17] performed a fusionless hemilaminectomy using the unilateral approach on 26 patients with multiple-level compressive cervical spinal stenosis. No deterioration was observed in the sagittal balance of the cervical spine during long-term patient follow-up. With the recent developments of more specialized instruments and devices, minimally invasive spinal surgery has been demonstrated to be a useful procedure for treating spinal diseases while also minimizing the damage to soft tissues. The posterior minimally invasive cervical approach is being used in many institutions to determine the feasibility and efficacy of such procedures. In a recent study, Hernandez et al. [18] using a tubular retractor to perform minimally invasive decompression for radiculopathy and myelopathy confirmed that the basic technique was safe and feasible. Visualization of the spinal canal, ligamentum flavum, and exiting nerve root interface is facilitated via a surgical microscope that provides a three-dimensional view. In our technique, decompression can be performed without the need for a tubular retractor. It is an alternative to the minimally invasive technique. Using this microscope-assisted procedure, we could successfully perform bilateral decompression using the unilateral approach with hemi-partial laminectomy, also known as unilateral approach and bilateral decompression (ULBD).

Conventional laminectomy causes cervical instability and kyphosis when facet joints are resected [19,20]. Dynamic X-rays should be performed to rule out any instability, and the scope of surgery should be determined via MRI or computed tomography. Our minimally invasive approach using the ULBD technique, which requires only hemi-partial laminectomy to widen the spinal canal, can minimize facet joint resection. Moreover, the hospital stay duration was relatively shorter in our patient compared with patients reported in published data on conventional open surgeries. In summary, disruption of the anatomic integrity of posterior structures leads to changes in the capacity of the spine for physiological movement, thereby increasing the risk of developing kyphosis or other deformities. The technique presented herein is a modification of the standard laminectomy procedure. We used this technique because it is simple, does not require fixation, and does not significantly damage the associated muscle attachments or facet joints. In contrast to other minimally invasive techniques that are performed with hemilaminectomy, in our technique decompression can be achieved with hemi-partial laminectomy.

Our technique has two basic advantages over standard laminectomy: 1. it preserves the biomechanical functions of the posterior muscle complex with minimal skin incisions and 2. it maintains joint integrity via partial laminectomy. The reported technique protects the neural arc, which provides stability to the cervical spine and spinous processes to which some parts of the cervical muscle complex attach. Despite the many advantages, our technique is appropriate for only selected patients with posteriorly originated hypertrophy or ossification of the ligamentum flavum that is not accompanied by anteriorly originated spinal stenosis.

## Conclusions

Conclusion

This minimally invasive posterior approach can be safely applied as an alternative to classic laminectomy to relieve pain and neurological dysfunctions in myelopathy caused by posteriorly-originated compressive spinal stenosis. The technique has many advantages, such as shorter hospital stay, less postoperative pain, and decreased morbidity compared with the standard laminectomy and laminoplasty techniques. It also has important advantages of preserving the neural arc, partial hemilaminectomy, and limited muscle dissection. These advantages include less postoperative pain medication and quicker return to resuming daily life.
